# Preparation of Patient for Abdominal Section and Treatment of the Case1Read before the Saint Louis Medical Society, December 5, 1903.

**Published:** 1904-09

**Authors:** Walter B. Dorsett

**Affiliations:** Saint Louis


### SELECTIONS.

### Preparation of Patient for Abdominal Section and Treatment of the Case.[1]

By WALTER B. DORSETT, M. D., Saint Louis.

[From *Saint Louis Medical Review*, January 23, 1904.]

Realising that medical literature is rather scant in the exposition of the subject matter of this text, and that what is to be gleaned is at the expense of a great deal of work (for it is found only as fragments), I have thought it would be instructive for this society to learn from our own members just what is the practice that is now considered orthodox. In order, therefore, to elicit a discussion I have taken the liberty to present my own views on this subject.

Faulty and imperfect preparation for a celiotomy often places a patient in a handicapped position to undergo the mental and physical ordeal that has to be encountered; so also does a careless and imperfect after-treatment unnecessarily jeopardise the life of the patient.

---

1. Read before the Saint Louis Medical Society, December 5, 1903.

From the birth of antiseptic and aseptic surgery to the present day many methods of preparation of the patient have been tried and found wanting, and in many cases disastrous results have been attributed to other causes than that of preparation and after-treatment; when in reality the cause lay here.

Frequently the methods in vogue have been lauded heavenward for the time, only to be entirely discarded or so modified as to be unrecognisable. As for instance, not a few members of this society will remember, when serving as internes in our City Hospital not many years ago, not to use a steam spray, reeking with carbolic acid, upon the intestines of a celiotomy patient was tantamount to a dismissal from the service. Today such treatment would be most vigorously condemned.

Following close upon the heels of the carbolic spray came the bichlorid pack, which was applied to the abdomen for from six to twelve hours prior to the operation. This was often as strong as 1 to 1000 or stronger, and not infrequently when the patient came to the table, distinct vesication of the skin could be seen. So it will be seen that during the evolution of this effort at cleanliness many, many changes necessarily took place.

A careful retrospection of the subject will convince one that the tendency is toward simplicity and cleanliness, rather than complexity and antisepsis.

The use of chemical irritants in and around the field of operation has been supplanted by good clean soap and hot water. There is no question in my mind that the irritation incident to the scrubbing with a stiff brush with a solution of corrosive sublimate is positively dangerous to the patient. The protective epithelium is scraped and torn off by the coarse, rough brush that is usually used, and while the bichlorid solution may destroy organisms here situated, the part is left in a far more suitable state for the propagation of pathogenic organisms than before it was touched.

A good scrubbing with green soap and sterile hot water with a soft brush, or perhaps better still, a Turkish bath towel, followed by the application of the razor, to not alone remove the hair, but to scrape off excretions of the skin that have been brought to the surface by the soap and water, is all that is necessary until the patient is brought to the table.. The part may be protected by a thick layer of sterilised gauze or absorbent cotton which is allowed to remain in place by bandage until the patient is anesthetised and brought to the operating-room. This is then removed and the site again scrubbed or rather wiped off with a solution of green soap, then rinsed off with sterile water, after which sulphuric ether is applied, and when it has disappeared by evaporation, alcohol is applied. Sterile towels are then applied around the proposed incision, taking care not to allow too great an area to be uncovered. During the operation the surgeon should avoid touching the skin of the patient unnecessarily, and at no time, if the intestines be brought through the incision, should they ever be allowed to rest upon the skin, but upon sterile towels, which should be changed as soon as soiled.

### CATHARSIS.

From observation of my own cases and others I am inclined to believe that in our zeal to get the intestinal tract free from fecal matter, hypercatharsis is obtained to the great detriment of the patient's strength. Particularly is this true in the use of epsom salts in large doses. This applies particularly to cases in which quite an amount of blood may be lost, as in hysterectomies, myomectomies or salpingotomies, and the like. My favorite formula for all celiotomies is as follows:

| | | |
|---|---|---|
| ℞ | Magnes. sulph. | dr. iii |
| | Sol. magnes. citrate | oz. xii. |
| | Sol. carmin | m. xv. |

Sig. One-third contents of bottle every three or four hours till bowels act.

In most instances one or two parts of this solution is all that is necessary. On the morning of the operation and two or three hours prior thereto, a copious soapsuds enema is given for the purpose of the removal of the remaining fecal matter, so that should it be desired to give a copious saline enema in case of shock from great loss of blood or otherwise, you have a better absorbing surface in the colon.[1]

### THE PREVENTION OF THE EMESIS OF ANESTHESIA.

Post operative emesis is the "bete noir" of the abdominal surgeon. This distressing condition often lasts for hours and even days, and its dangers are often far-reaching. It weakens the heart's action, disturbs wound coaptations, not only in abdominal incision but wounds in the repaired abdominal viscera as well, to say nothing of its production of gastric inflammation, which in many instances becomes a more serious condition than that for which the operation was performed. Again, this condition of vomiting is not always easily differentiated from intestinal obstruction.

Aside from the fasting for twelve hours before the operation and the post operative inhalation of vinegar or acetic acid,

---

1. As to the specific action of magnesium sulphate, I will say that from my own observation in a case of biliary fistula at the Female Hospital some twelve years ago, I am satisfied that by its administration the secretion of the liver is very materially stimulated, and reasoning by analogy I am led to believe that other secretory organs are likewise thus stimulated.

nothing was done for my patients to prevent this distressing condition until a short time ago, when I chanced to see a short note in a journal on the cause of chloroform vomiting and the cold water treatment therefor. I immediately called to mind several cases in which I had been greatly worried as to this condition and I resolved to try the treatment. The author, in this note, calls to mind the observance of the action of the muscles of deglutition which we frequently notice in the patient while the anesthetic is being administered. The mask is well applied over the nose or mouth, or both, and the anesthetic is poured or dropped on. After two or three inhalations, if notice is taken, the patient will be seen to swallow as well as inhale, and the vapor of the anesthetic is taken into the stomach more rapidly perhaps than into the air passages. The stomach is empty and the superimposed walls are separated and the anesthetic comes in direct contact with the gastric mucous membrane, with the result of producing not alone an irritation but an inflammation. Thus is a gastritis produced and as a consequence vomiting as a reflex neurotic symptom established.

Now, by allowing the patient to drink from thirty to forty ounces of water in quantities of ten ounces at intervals of a half hour prior to going to the operating room, the stomach is filled with the water, and as soon as the chloroform or ether enters the stomach it is absorbed by the water and is held in solution, and by this dilution it cannot irritate the gastric walls. The last ten ounces are given immediately before the anesthetist begins the anesthesia, so that should the water taken prior to this last quantity be absorbed, this last portion of water, not having had the time to be absorbed, readily takes up the vapor. We have observed in most instances this last portion is vomited with but little effort, and the nausea here ceases. So far this practice has greatly lessened my anxiety in many instances.[1]

### THE DRESSING OF THE ABDOMINAL INCISION.

Much as been written on the "abdominal incision," "the through and through suturing," "suturing in layers," and the like, all having as their aim thorough coaptation and elimination of dead spaces. But little has been said as to the best method of maintaining this coaptation until nature in her reparative process has accomplished her purpose.

Ventral hernias are apt to follow and do follow the work of our best men, whether one or the other method of suturing is practised. I take it that this is due to the fact that little or no

1. The rationale of lavage for emesis is probably the solution of chloroform from the gastric walls brought or siphoned from the stomach by means of the stomach tube.

attempt is made to combat intraäbdominal pressure, which is aggravated by the coughing and vomiting.

To prevent this I am in the habit of folding pads of gauze very tightly so as to make them hard, applying them on each side of and over the line of incision, and maintaining them in contact with the abdominal wall by a larger piece of gauze that is sealed down with collodion. This firm dressing when the binder is drawn snugly, acts as a buffer, or in the same manner as does the pad of a truss in hernia.

The collodion also prevents the dressing from slipping off the wound during convalescence when the patient turns on her side. It is not removed until the tenth day after the operation, when the stitches are removed.

### GASEOUS DISTENTION.

One of the most annoying conditions in the treatment of the abdominal patient is the distention of the abdomen with gas. In order to forestall this trouble some surgeons are in the habit of making an effort towards opening the bowels by the administration of laxatives on the day following the operation. This I believe is a mistake, inasmuch as the excitation of peristaltic action of the entire intestinal tract by medicines so disturbs relations of parts as to hinder union of surfaces, and in pus cases spreads infection that would otherwise remain local. In order to relieve this condition, which in the majority of cases is a paresis, I have been using the Virgil O. Hardon treatment—namely, enemata of alum solution, which is one-half ounce of pulverised alum to one pint of warm water, carried well into the colon by means of the colonic tube, best introduced with the patient in the left lateral or Sims position. While alum is ordinarily regarded as an astringent, the amount of peristalsis of the colon it produces is remarkable. By the peristalsis of the colon the distention, which ordinarily is in the ileum, is relieved through the ileo-cecal opening into the colon and the gas is expelled. A 50 per cent. solution of fresh ox gall is advocated by Ameiss and the formula now in use at St. Mary's Infirmary, and probably originated by Dr. McCandless, is:

| | | |
|---|---|---|
| ℞ | Ox gall | dr. ss. |
| | Turpentine | dr. ss. |
| | Saturated solution magnes. sulph | oz. iv. |
| | Glycerin | oz. iv. |
| | Aquæ puræ, q. s. | O. i. |

I have used with varied success, but so far I prefer the solution of alum as proposed by Hardon.

